# Dose Intensity/Body Surface Area Ratio is a Novel Marker Useful for Predicting Response to Lenvatinib against Hepatocellular Carcinoma

**DOI:** 10.3390/cancers12010049

**Published:** 2019-12-22

**Authors:** Yuji Eso, Shigeharu Nakano, Masako Mishima, Soichi Arasawa, Eriko Iguchi, Fumiyasu Nakamura, Haruhiko Takeda, Atsushi Takai, Ken Takahashi, Kojiro Taura, Hiroshi Seno

**Affiliations:** 1 Department of Gastroenterology and Hepatology, Graduate School of Medicine, Kyoto University, 54 Shogoin-Kawaharacho, Sakyo-ku, Kyoto 606-8507, Japan; shnakano@kuhp.kyoto-u.ac.jp (S.N.); masakoiw@kuhp.kyoto-u.ac.jp (M.M.); sarasawa@kuhp.kyoto-u.ac.jp (S.A.); iguchi@kuhp.kyoto-u.ac.jp (E.I.); nakamura@kuhp.kyoto-u.ac.jp (F.N.); htakeda@kuhp.kyoto-u.ac.jp (H.T.); atsushit@kuhp.kyoto-u.ac.jp (A.T.); takaken@kuhp.kyoto-u.ac.jp (K.T.); seno@kuhp.kyoto-u.ac.jp (H.S.); 2 Department of Surgery, Graduate School of Medicine, Kyoto University, 54 Shogoin-Kawaharacho, Sakyo-ku, Kyoto 606-8507, Japan; ktaura@kuhp.kyoto-u.ac.jp

**Keywords:** ALBI grade, body surface area, dose intensity, hepatocellular carcinoma, lenvatinib, molecular-targeted therapy, relative dose intensity

## Abstract

Lenvatinib was recently approved as a novel first-line molecular targeted agent (MTA) for treating hepatocellular carcinoma (HCC). The importance of relative dose intensity (RDI) has been shown in the treatment of various types of cancers. However, RDI may not accurately reflect the treatment intensity of lenvatinib, as it is the first oral MTA where the dose is based on the patient’s weight. We aimed to evaluate the utility of 2M-DBR (the delivered dose intensity/body surface area ratio at 60 days) by comparing the relationship between 2M-DBR, 2M-RDI (RDI at 60 days), and the therapeutic response. The therapeutic response to lenvatinib was evaluated in 45 patients who underwent computed tomography 8–12 weeks after treatment initiation. We also investigated the clinical factors associated with high 2M-DBR. The area under the receiver operating characteristic of 2M-DBR that predicts the response to lenvatinib was higher than that of 2M-RDI (0.8004 vs. 0.7778). Patients with high 2M-DBR achieved significantly better objective responses and disease control rates than those with low 2M-DBR (*p* < 0.0001 and 0.0008). Patients with high 2M-DBR experienced significantly longer progression-free survival (PFS) than those with low 2M-DBR (*p* = 0.0001), while there was no significant correlation between 2M-RDI levels and PFS (*p* = 0.2198). Patients who achieved higher levels of 2M-DBR had a significantly better modified ALBI grade (*p* = 0.0437), better CONUT score (*p* = 0.0222), and higher BTR (*p* = 0.0281). Multivariate analysis revealed that high 2M-DBR was the only significant factor associated with longer PFS. In conclusion, 2M-DBR could be an important factor that reflects treatment intensity and useful for predicting the response to lenvatinib against HCC, instead of 2M-RDI.

## 1. Introduction

Hepatocellular carcinoma (HCC) remains one of the leading causes of cancer-related deaths globally [1]. For patients with advanced-stage HCC, systemic chemotherapy using molecular targeted agents (MTA) has been the recommended standard treatment [2,3]. Sorafenib was approved in 2007 as the first MTA that demonstrated a significant survival benefit in patients with unresectable HCC based on the two Phase III studies [4,5]. However, due to its various adverse events (AEs) and limited efficacy, identification of novel MTA compounds has been required. Recently, lenvatinib (Lenvima^®^, Eisai Co., Ltd., Tokyo, Japan) was approved as a novel first-line MTA for unresectable HCC in the EU, USA, and Asia, including Japan, China, Korea, and Taiwan, based on the Phase III REFLECT trial [6].

In order to optimize and maximize the therapeutic effects of pharmacotherapy, especially in the case of anticancer drug treatment, it is essential to appropriately evaluate the patient’s condition before administration, sufficiently manage AEs after initiation, and keep the dose intensity (DI) high. The practicality of relative dose intensity (RDI), calculated as the percentage of the delivered DI divided by the standard DI, has been shown to be an indicator of the treatment intensity of anticancer drugs [7]. The correlations between RDI and therapeutic efficacy were reported in various types of cancers, including breast cancer [8], pancreatic ductal adenocarcinoma [9], renal cell carcinoma [10], malignant lymphoma [11], and HCC [12]. However, since lenvatinib is the first oral MTA that is dosed by the patient’s weight (8 mg once a day for patients weighing <60 kg or 12 mg once a day for those weighing ≥60 kg), RDI may not accurately reflect the actual treatment intensity, as is demonstrated in the following examples.

Case 1: A 170 cm tall patient weighing 60 kg. He started lenvatinib at 12 mg once a day and continued to take 12 mg once a day for the first 20 days. In the next 40 days, lenvatinib was reduced to 8 mg once a day due to AEs.

RDI at 60 days (2M-RDI) = 560 (12 mg × 20 days + 8 mg × 40 days)/720 (12 mg × 60 days) = 0.78 (78%). (1)

Case 2: A 170 cm tall patient of 59 kg. He started lenvatinib at 8 mg once a day and continued with 8 mg for 60 days.

2M-RDI = 480 (8 mg × 60 days)/480 (8 mg × 60 days) = 1.00 (100%). (2)

Cases 1 and 2 are patients of almost the same weight, and Case 1 was administered with a higher delivered DI compared to Case 2. However, there is a contradiction in the 2M-RDI between Cases 1 and 2. On the other hand, when the delivered DI/body surface area (BSA) ratio at 60 days (2M-DBR) after lenvatinib induction is calculated in the same examples, then:

Case 1: 2M-DBR = 560 (12 mg × 20 days + 8 mg × 40 days)/1.695 m^2^ = 330.39, (3)

Case 2: 2M-DBR = 480 (8 mg × 60 days)/1.683 m^2^ = 285.23. (4)

Therefore, we hypothesized that 2M-DBR might reflect treatment intensity of lenvatinib more accurately than 2M-RDI. This study aimed to evaluate the utility of 2M-DBR by comparing the relationship between 2M-DBR or 2M-RDI and the therapeutic efficacy. We also investigated the clinical factors and biomarkers that were associated with 2M-DBR.

## 2. Results

### 2.1. Baseline Characteristics of Patients

The baseline clinical characteristics of enrolled patients are shown in Table 1. The study included 37 males and 8 females (mean age, 70.4 years; age range, 50–85 years). Thirty patients weighed ≥60 kg, and 15 patients weighed <60 kg. There were 6 patients with hepatitis B virus (HBV), 16 with hepatitis C virus (HCV), and 23 with hepatitis of non-B non-C. Nucleot(s)ide analogues were administered to 4 patients with HBV infection. Eight patients with HCV-related hepatitis had achieved sustained viral response by direct-acting agents or interferon-based therapy. There were 16 patients with a TNM stage of III, 8 with stage IV A, and 21 with stage IV B. Five patients started lenvatinib as an initial treatment, while 40 patients had a treatment history of HCC. There were 23 patients with a Child–Pugh score of 5, 16 patients with a Child–Pugh score of 6, and 6 patients with a Child-Pugh score of 7. Regarding an initial dose of lenvatinib, 17 patients started with 12 mg, 25 patients started with 8 mg, and 3 patients started with 4 mg. Twenty-nine patients started lenvatinib with the standard dose, while 16 patients started lenvatinib with a reduced dose.

### 2.2. Therapeutic Response to Lenvatinib According to 2M-RDI or 2M-DBR

Among 45 patients, 25 patients experienced down-dosing, and 18 patients experienced a temporary interruption of lenvatinib during the first 60 days. The median 2M-RDI and 2M-DBR were 66.7% (range, 11.7–100%) and 241.9 (range, 34.1–432.5), respectively. According to the results of the dynamic contrast-enhanced computed tomography (CECT) scans evaluated at 8–12 weeks, complete response (CR) was noted in 3 patients, partial response (PR) in 15, stable disease (SD) in 10, and progressive disease (PD) in 17 patients following the modified Response Evaluation Criteria in Solid Tumors (mRECIST) guidelines. The objective response rate (ORR, CR + PR) and the disease control rate (DCR, CR + PR + SD) at 8–12 weeks were 40.0% and 62.2%, respectively (Table 2).

**Figure 1:**
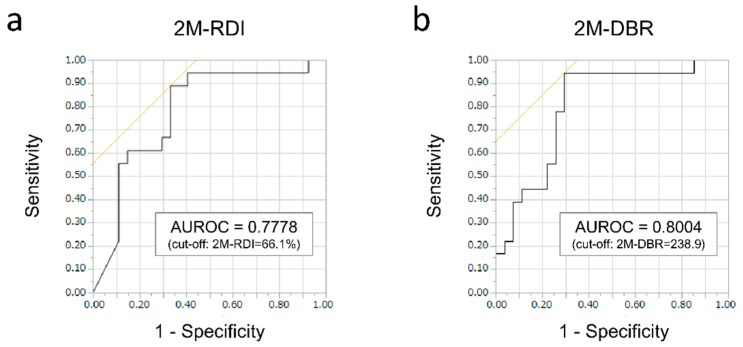
Receiver operating characteristic (ROC) curve analyses of the relative dose intensity at 60 days (2M-RDI) and the delivered dose intensity/body surface area ratio at 60 days (2M-DBR) to predict the objective response to lenvatinib at 8–12 weeks. (**a**) The area under the ROC (AUROC) of 2M-RDI was 0.7778 at an optimal cut-off value of 66.1% (sensitivity, 88.9%; specificity, 66.7%). (**b**) The AUROC of 2M-DBR was 0.8004 at an optimal cut-off value of 238.9 (sensitivity, 94.4%; specificity, 70.4%), which was higher than that of 2M-RDI.

**Figure 2:**
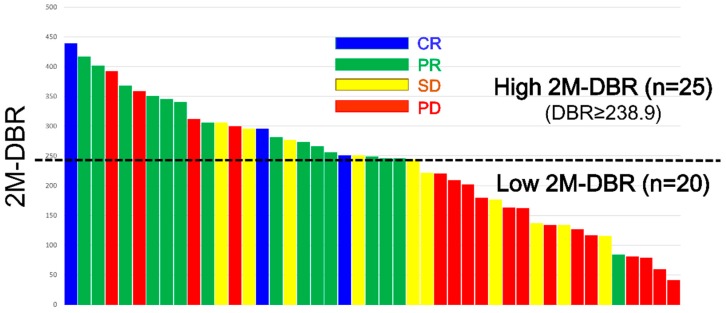
Responses to lenvatinib of the 45 enrolled patients were evaluated by contrast-enhanced computed tomography at 8–12 weeks, which was sorted by the delivered dose intensity/body surface area ratio at 60 days (2M-DBR).

In order to select the cut-off values that differentiate responders from non-responders, we performed receiver operating characteristic (ROC) curve analyses of 2M-RDI and 2M-DBR to predict the objective response (CR or PR), where we compared the area under the ROC (AUROC) among them. As a result, the AUROC of 2M-RDI was 0.7778 at an optimal cut-off value of 66.1% (sensitivity, 88.9%; specificity, 66.7%; Figure 1a), while the AUROC of 2M-DBR was 0.8004 at an optimal cut-off value of 238.9 (sensitivity, 94.4%; specificity, 70.4%; Figure 1b), which was higher than that of 2M-RDI. When comparing the ORR and DCR, divided into two groups according to the 2M-DBR levels, the ORR and DCR of the high 2M-DBR group (≥238.9, *n* = 25) were 68.0% and 84.0% (CR in 3, PR in 14, SD in 4, and PD in 4), respectively, which were significantly higher than those of the low 2M-DBR group (<238.9, *n* = 20; 5.0% and 35.0%) (*p* < 0.0001 and *p* = 0.0008, see Table 3 and Figure 2).

### 2.3. Adverse Events Related to Lenvatinib Therapy

AEs related to lenvatinib therapy is shown in Table 4. Hypertension was the most common AE in the present study (Any grade: 55.6%, *n* = 25; Grade ≥3: 15.6%, *n* = 7), followed by general fatigue (Any grade: 46.7%, *n* = 21), diarrhea (Any grade: 35.6%, *n* = 16), appetite loss (Any grade: 33.3%, *n* = 15), hand–foot skin reaction (Any grade: 26.7%, *n* = 12), weight loss (Any grade: 24.4%, *n* = 11), proteinuria (Any grade: 22.2%, *n* = 10), hypothyroidism (Any grade: 22.2%, *n* = 10), and hoarseness (Any grade: 15.6%, *n* = 7).

### 2.4. Relationship Between 2M-RDI or 2M-DBR and Progression-Free Survival

**Figure 3:**
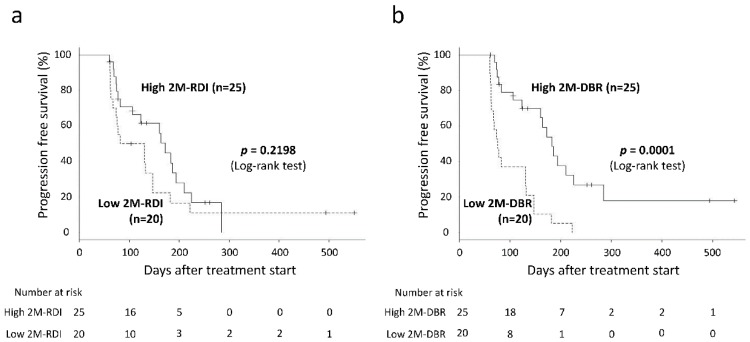
Progression-free survival (PFS) according to the relative dose intensity at the 60 day (2M-RDI) levels or the delivered dose intensity/body surface area ratio at 60 days (2M-DBR). (**a**) No significant difference was noted in PFS between the high 2M-RDI group and the low 2M-RDI group (log-rank test, *p* = 0.2198). (**b**) PFS of the high 2M-DBR group was significantly longer than that of the low 2M-DBR group (log-rank test, *p* = 0.0001).

The median progression-free survival (PFS) of all enrolled patients was 123.0 days (range, 60–551 days). When PFS was compared according to the 2M-RDI levels, there was no significant difference between the PFS of the high 2M-RDI group (≥66.1%, *n* = 25) and that of the low 2M-RDI group (<66.1%, *n* = 20) (log-rank test, *p* = 0.2198, Figure 3a). On the other hand, the PFS of the high 2M-DBR group was significantly longer than that of the low 2M-DBR group (log-rank test, *p* = 0.0001, Figure 3b).

### 2.5. Comparison between Patients with High 2M-DBR and Low 2M-DBR

Next, we compared the baseline characteristics between the high 2M-DBR group and the low 2M-DBR group. Patients with high 2M-DBR had significantly higher BSA (*p* = 0.0309), a better modified albumin-bilirubin (mALBI) grade (*p* = 0.0437), a higher branched-chain amino acids tyrosine ratio (BTR, *p* = 0.0222), and a better Controlling Nutrition Status (CONUT) score (*p* = 0.0281), indicating that well-preserved liver function and favorable nutritional status before treatment are essential for achieving high 2M-DBR levels of lenvatinib (Table 5).

### 2.6. Factors Associated with Progression-Free Survival

Finally, we investigated the factors associated with longer PFS in lenvatinib therapy by univariate and multivariate analyses. The Cox proportional hazards model showed that baseline BTR and high 2M-DBR were associated with longer PFS, as per the univariate analyses (*p* = 0.0401 and *p* = 0.0003, respectively). Multivariate analysis revealed that high 2M-DBR was the only significant factor associated with longer PFS (hazard ratio (HR) = 0.29, 95% CI = 0.11–0.76; *p* = 0.0127) (Table 6).

## 3. Discussion

Lenvatinib was recently approved as a novel first-line tyrosine kinase inhibitor for unresectable HCC based on the Phase III REFLECT trial [3,6]. The Phase II study performed before the REFLECT trial revealed positive outcomes with an overall survival (OS) of 18.7 months, ORR of 37%, and DCR of 78% [13]. The dosage of lenvatinib in the Phase II study was set to 12 mg once a day until the disease progression or the unacceptable toxicity was evident based on the results of the Phase I dose-escalation study in 20 patients [14,15]. In the Phase II study, however, many patients required dose reduction (74%) or drug discontinuation (22%) due to AEs. Therefore, based on these results and pharmacokinetic analysis, the Phase III REFLECT trial proceeded with a planned dose of 8 mg once a day in patients weighing <60 kg and 12 mg once a day in those weighing ≥60 kg [15]. As a result, lenvatinib met its primary endpoint by demonstrating a prolonged effect on OS as confirmed by non-inferiority to sorafenib, and led to a statistically significant improvement for all secondary efficacy endpoints, including PFS, time to progression, and ORR [6]. Dose reductions and drug discontinuations due to AEs were needed for 37% and 9%, respectively, which decreased compared with those in the Phase II study.

It is well accepted that RDI is a useful indicator for evaluating the feasibility of pharmacotherapy, especially anticancer drug treatment [7]. The importance of RDI has also been reported in molecular targeted therapy for HCC [12,16]. Wang et al. investigated the significance of RDI for the first month (1M-RDI) of regorafenib in patients with HCC, which reported that patients with 1M-RDI ≥50% showed significantly longer OS and PFS than patients with 1M-RDI <50% [12]. The standard dose of regorafenib for HCC is 160 mg orally once a day, regardless of body weight [17]. On the other hand, as mentioned earlier, the standard dose of lenvatinib is normally determined by the patient’s weight; therefore, in order to accurately reflect the treatment intensity, another index is required besides the RDI.

In the present study, we demonstrated for the first time that 2M-DBR could reflect the treatment intensity of lenvatinib against HCC more accurately than 2M-RDI. The AUROC of 2M-DBR in predicting the objective response to lenvatinib on CECT at 8–12 weeks after starting treatment was higher than that of 2M-RDI (0.8004 vs. 0.7778). Patients with high 2M-DBR levels achieved significantly better ORR and DCR than those with low 2M-DBR (*p* < 0.0001 and *p* = 0.0008). Furthermore, patients with high 2M-DBR experienced significantly longer PFS than those with low 2M-DBR (log-rank test, *p* < 0.0001), while there was no significant correlation between 2M-RDI levels and PFS. Patients who achieved high 2M-DBR levels had a significantly higher BSA (*p* = 0.0309), a better baseline liver function (mALBI grade (*p* = 0.0437)), and a better nutritional status (CONUT score (*p* = 0.0281) and BTR (*p* = 0.0222)). In univariate analyses, the baseline BTR level and high 2M-DBR were correlated with longer PFS. Multivariate analysis revealed that high 2M-DBR was the only significant factor associated with longer PFS (*p* = 0.0127). Taken together, 2M-DBR is an important factor in reflecting treatment intensity and predicting the response to lenvatinib, where the pretreatment liver function and nutritional status are essential for achieving high 2M-DBR levels. Using 2M-DBR will help to develop an appropriate lenvatinib treatment strategy tailored to the patient. From this study, it was shown that lenvatinib achieves a high probability of objective response (CR or PR) and longer PFS if 2M-DBR was 238.9 or higher. Therefore, by calculating the target dose of 60 days (2M-DBR ≥ 238.9) for each patient before the treatment starts, it becomes possible to establish a treatment strategy that balances treatment effects and AE management through an appropriate dosage adjustment after treatment starts.

Hiraoka et al. previously reported that the mALBI grade was the only significant prognostic factor in HCC patients treated with lenvatinib [18]. Ueshima et al. also recently published the data of 82 HCC patients, which demonstrated that patients with an mALBI grade of 1 had the lowest probability of treatment discontinuation due to AEs (*p* < 0.01) [19]. They also showed that an mALBI grade of 1 was a significant predictor of a high ORR (*p* < 0.05) [19]. In agreement with these reports, we confirmed that the baseline mALBI grade was essential for obtaining high 2M-DBR levels. Interestingly, we also identified, for the first time, that the pretreatment nutritional status, which was measured by the CONUT score and BTR, was essential for achieving high 2M-DBR. The utility of the CONUT score in predicting the patient’s prognosis treated with chemotherapy was reported in various types of gastrointestinal cancers [20,21,22]. Although there was no significant correlation between the CONUT score and PFS in lenvatinib treatment in our study (*p* = 0.1505), it may be worth reconsidering in a more extensive cohort study. The impact of BTR as a prognostic factor in patients with HCC or liver cirrhosis was well documented [23,24,25]. Tada et al. recently reported that BCAA therapy improved both OS and disease-specific survival in HCC patients with low BTR levels [25]. Further studies with the prospective design are required to clarify the utility of BCAA therapy during treatment with lenvatinib.

Our findings provide important clinical implications for the feasibility of 2M-DBR in assessing treatment intensity and predicting the response to lenvatinib against HCC. However, our study has some limitations. First, our study had a retrospective and single-center design with a limited sample size. Therefore, the possibility of selection bias cannot be denied. Second, we could not evaluate whether 2M-DBR has an impact on OS due to the limited sample size and relatively short observation period. Additionally, the optimal cut-off value and the setting period of DBR should be investigated in a large cohort study. Thus, in interpreting our findings, caution should be exercised. Further studies are desired to validate our observations and investigate the relationship between 2M-DBR and the therapeutic efficacy of lenvatinib, including ORR, DCR, PFS, and OS.

## 4. Materials and Methods

### 4.1. Study Design

A total of 49 patients with unresectable HCC received treatment with lenvatinib at Kyoto University Hospital (Kyoto, Japan) from March 2018 to September 2019. Among them, 45 patients, who underwent dynamic CECT 8–12 weeks after treatment initiation, were included in this study to evaluate the therapeutic response to lenvatinib. HCC was diagnosed based on the criteria of the EASL practice guideline [2]. We collected the clinical data regarding these patients prior to treatment with lenvatinib, including body weight, BSA, treatment history, hepatitis virus-related markers, liver function markers (aspartate aminotransferase, alanine aminotransferase, platelet count, serum albumin (ALB), total bilirubin, prothrombin time, Child–Pugh score/grade, albumin-bilirubin (ALBI) score, mALBI grade), nutritional status markers (BTR, CONUT score), liver fibrosis markers (fibrosis-4 (FIB-4) index, Mac-2 binding protein glycosylation isomers (M2BPGi), tumor markers (α–fetoprotein, des-γ-carboxy prothrombin), and tumor burdens that were determined by CECT scan. BSA was calculated using the Du Bois formula: 0.007184 × Height^0.725^ × Weight^0.425^. The ALBI score and mALBI grade were calculated as previously reported [19,26]. The CONUT score was calculated using the serum ALB, total cholesterol, and total lymphocyte count as was described previously [27,28]. The FIB-4 index was calculated by age (years) × AST (IU/L)/PLT counts (×10^9^/L) × √ALT (IU/L) [29], while M2BPGi levels were measured using the HISCL M2BPGi kit (Sysmex, Hyogo, Japan) [30]. The tumor node metastasis (TNM) stage proposed by the American Joint Committee on Cancer/Union for International Cancer Control was used for the evaluation of tumor progression. The ethics committee of Kyoto University Hospital approved the protocol of this study (R1740). This study protocol conformed to the ethical guidelines of the Declaration of Helsinki.

### 4.2. Protocol of Treatment with Lenvatinib

The initial dose of lenvatinib was 8 mg once a day to patients weighing <60 kg and 12 mg once a day to those weighing ≥60 kg. However, at the discretion of the attending physician, a reduction of the initial dose was allowed after obtaining informed consent. According to the guidelines for administration of lenvatinib provided by the manufacturer, the dose of lenvatinib was reduced, or the treatment was interrupted, when the patient experienced any unacceptable drug-related AEs. AEs were assessed according to the National Cancer Institute Common Terminology Criteria for Adverse Events, version 4.0. According to the administration guideline provided by the manufacturer, dose reduction or temporary interruption of lenvatinib was maintained until the AEs were resolved to Grade 1 or 2.

### 4.3. Calculation of 2M-RDI and 2M-DBR

2M-RDI was calculated as the percentage of delivered DI (total delivered dose for the first 60 days) divided by the standard DI of lenvatinib for 60 days. The standard DI of lenvatinib for 60 days was as follows: 480 mg (8 mg × 60 days) in patients weighing <60 kg and 720 mg (12 mg × 60 days) in patients weighing ≥60 kg.

2M-DBR was calculated as the delivered DI for the first 60 days divided by BSA. BSA was calculated based the patient’s height and body weight just before treatment initiation of lenvatinib.

### 4.4. Evaluation of Therapeutic Response

Dynamic CECT scans were carried out every 8–12 weeks after treatment initiation to evaluate the therapeutic response to lenvatinib. The response to lenvatinib (CR, PR, SD, or PD) was evaluated by investigators following the mRECIST guidelines [31].

### 4.5. Statistical Analyses

ROC curve analyses were performed to calculate the AUROC and selecting the optimal cut-off value that maximized the sum of both the sensitivity and specificity (Figure 1). Survival curves were created using the Kaplan–Meier method and compared by the log-rank test (Figure 3). The differences in categorical variables between the groups were analyzed using the Pearson’s chi-square test for categorical variables (Table 3; Table 5), Welch’s *t*-test for continuous variables that showed a normal distribution (ALB and prothrombin time in Table 5), or Mann-Whitney’s *U* test for continuous variables that did not show a normal distribution (Age, BSA, total bilirubin, ALBI score, BTR, CONUT score, FIB-4 index, and M2BPGi in Table 5). A Cox proportional hazards model was used to detect the HR for univariate and multivariate analyses (Table 6). The PFS was measured from the date of lenvatinib administration to the date of radiological tumor progression or death from any cause. A *p*-value of < 0.05 was considered statistically significant. Statistical analyses were performed using JMP^®^ Pro 14 for Windows (SAS Institute, Cary, NC, USA) or Easy R (EZR), a graphical user interface for R (The R Foundation for Statistical Computing, Vienna, Austria) [32].

## 5. Conclusions

We demonstrated, for the first time, that 2M-DBR could be a significant factor reflecting treatment intensity that could be useful for predicting the response to lenvatinib in patients with HCC. In lenvatinib therapy, pretreatment liver function as well as a favorable nutritional status is essential for achieving high 2M-DBR levels, while every effort should be made to minimize dose reductions through the appropriate management of AEs.

## Figures and Tables

**Table 1:** Baseline characteristics of patients.

Variable	*n* = 45
Age (years, range)	70.4 ± 8.61 (50–85)
Gender (Male vs. Female)	37/8
Body weight (<60kg vs. ≥60kg)	15/30
Body surface area (m^2^)	1.72 ± 0.17
Etiology (HBV vs. HCV vs. non-B non-C)	6/16/23
TNM stage (III vs. IVA vs. IV B)	16/8/21
BCLC stage (B vs. C)	16/29
Treatment history (Naïve vs. recurrence)	
<Treatment prior to lenvatinib>	5/40
Surgery	4
Radiofrequency ablation	1
TACE	25
Sorafenib	9
Regorafenib	1
AST (IU/L)	44.3 ± 24.7
ALT (IU/L)	28.0 ± 15.0
Platelets (x10^4^/μL)	14.9 ± 7.00
ALB (g/dL)	3.56 ± 0.44
T-Bil (mg/dL)	0.90 ± 0.35
PT (%)	94.0 ± 15.5
Child–Pugh score (5A vs. 6A vs. 7B)	23/16/6
ALBI score	−2.27 ± 0.40
mALBI grade (1 vs. 2a vs. 2b vs. 3)	11/13/21/0
BTR	5.85 ± 2.17
CONUT score	3.64 ± 1.80
FIB-4 index	5.17 ± 4.30
M2BPGi (cut-off index)	2.87 ± 2.93
AFP (ng/mL)	8844 ± 27760
DCP (mAU/mL)	5183 ± 9891
Initial dose of lenvatinib (4 mg vs. 8 mg vs. 12 mg)	3/25/17
Initial dose of lenvatinib (standard vs. reduced)	29/16

Values are presented as mean ± standard deviation (range) or number. Abbreviations: HBV, hepatitis B virus; HCV, hepatitis C virus; BCLC, Barcelona Clinic Liver Cancer; TACE, transarterial chemoembolization; AST, aspartate aminotransferase; ALT, alanine aminotransferase; ALB, albumin; T-Bil, total bilirubin; PT, prothrombin time; ALBI, albumin-bilirubin; mALBI, modified ALBI; BTR, branched-chain amino acids tyrosine ratio; CONUT, Controlling Nutrition Status; FIB-4, Fibrosis-4; M2BPGi, Mac-2-binding protein glycosylation isomer; AFP, α–fetoprotein; DCP, des-γ-carboxy prothrombin.

**Table 2:** Response to lenvatinib at 8–12 weeks after lenvatinib induction.

Response	*n* = 45
Complete response (CR)	3
Partial response (PR)	15
Stable disease (SD)	10
Progressive disease (PD)	17
Objective response rate (ORR)	40.0%
	(18/45)
Disease control rate (DCR)	62.2%
	(28/45)

**Table 3:** Response to lenvatinib according to the 2M-DBR levels.

Response	High 2M-DBR Group (*n* = 25)	Low 2M-DBR Group (*n* = 20)	*p* Value
Complete response (CR)	3	0	
Partial response (PR)	14	1	
Stable disease (SD)	4	6	
Progressive disease (PD)	4	13	
Objective response rate (ORR)	68.0%	5.0%	<0.0001
	(17/25)	(1/20)	
Disease control rate (DCR)	84.0%	35.0%	0.0008
	(21/25)	(7/20)	

Abbreviations: 2M-DBR, dose intensity/body surface area ratio at 60 days.

**Table 4:** Adverse events (>10%).

Adverse Events	Any Grade (%)	Grade ≥3 (%)
Hypertension	25 (55.6)	7 (15.6)
General fatigue	21 (46.7)	2 (4.4)
Diarrhea	16 (35.6)	2 (4.4)
Appetite loss	15 (33.3)	2 (4.4)
Hand–foot skin reaction	12 (26.7)	1 (2.2)
Weight loss	11 (24.4)	1 (2.2)
Proteinuria	10 (22.2)	3 (6.7)
Hypothyroidism	10 (22.2)	1 (2.2)
Hoarseness	7 (15.6)	0

**Table 5:** Comparison between patients with high 2M-DBR and low 2M-DBR.

	High 2M-DBR Group (*n* = 25)	Low 2M-DBR Group (*n* = 20)	*p* Value
Age (years, range)	68.5 ± 8.47	72.8 ± 8.19	0.1018
Gender (male/female)	22/3	15/5	0.2226
Body weight (<60kg/≥60kg)	6/19	10/10	0.0702
BSA (m^2^)	1.76 ± 0.14	1.66 ± 0.18	**0.0309**
Etiology (HBV vs. HCV vs. non-B non-C)	5/8/12	1/8/11	0.3360
TNM stage (III vs. IVA vs. IV B)	9/3/13	7/5/8	0.4960
BCLC stage (B vs. C)	9/16	7/13	0.9445
Treatment history (Naïve vs. recurrence)	3/22	2/18	0.8320
AST (IU/L)	40.5 ± 19.9	49.0 ± 28.9	0.4172
ALT (IU/L)	27.0 ± 12.0	30.0 ± 18.8	0.9818
Platelets (×10^4^/μL)	15.0 ± 7.01	14.7 ± 6.95	0.9272
ALB (g/dL)	3.63 ± 0.50	3.49 ± 0.35	0.1385
T-Bil (mg/dL)	0.84 ± 0.25	0.98 ± 0.43	0.2316
PT (%)	92.1 ± 15.2	96.3 ± 15.7	0.7975
Child–Pugh score (5A vs. 6A vs. 7B)	14/9/2	9/7/4	0.4804
ALBI score	−2.33 ± 0.43	−2.18 ± 0.35	0.2007
mALBI grade (1 vs. 2a or 2b)	9/16	2/18	**0.0437**
BTR	6.78 ± 2.00	4.97 ± 1.94	**0.0222**
CONUT score	3.12 ± 1.70	4.30 ± 1.71	**0.0281**
FIB-4 index	4.67 ± 4.20	5.80 ± 4.33	0.2217
M2BPGi (cut-off index)	2.21 ± 2.61	3.60 ± 3.09	0.0760
Initial dose of lenvatinib(standard vs. reduced)	19/6	10/10	0.0702

Values are presented as mean ± standard deviation (range) or number. Abbreviations: 2M-DBR, dose intensity/body surface area ratio at 60 days; BSA, body surface area; HBV, hepatitis B virus; HCV, hepatitis C virus; BCLC, Barcelona Clinic Liver Cancer; TACE, transarterial chemoembolization; AST, aspartate aminotransferase; ALT, alanine aminotransferase; ALB, albumin; T-Bil, total bilirubin; PT, prothrombin time; ALBI, albumin-bilirubin; mALBI, modified ALBI; BTR, branched-chain amino acids tyrosine ratio; CONUT, Controlling Nutrition Status; FIB-4, Fibrosis-4; M2BPGi, Mac-2-binding protein glycosylation isomer.

**Table 6:** Comparison between patients with high 2M-DBR and low 2M-DBR.

Variable	No. of Cases	Univariate Analysis		Multivariate Analysis	
		HR (95% CI)	*p* Value	HR (95% CI)	*p* Value
Age, years	35/10	0.70 (0.34–1.53)	0.3507		
(≥64 vs. <64)					
Gender	8/37	1.69 (1.55–5.57)	0.2483		
(Female vs. Male)					
Body weight	30/15	0.64 (0.32–1.35)	0.2300		
(≥ 60kg vs. < 60kg)					
Body surface area, m^2^	29/16	0.53 (0.26–1.09)	0.0824		
(≥ 1.6623 vs. < 1.6623)					
ALB	9/36	0.45 (0.15–1.06)	0.0697		
(≥ 4.0 g/dL vs. < 4.0 g/dL)					
Child–Pugh score	22/23	1.32 (0.68–2.60)	0.4185		
(6 or 7 vs. 5)					
mALBI grade	11/34	0.51 (0.19–1.15)	0.1106		
(1 vs. 2a or 2b)					
BTR	8/25	0.36 (0.15–0.95)	**0.0401**	0.76 (0.27–2.18)	0.5971
(≥ 4.36 vs. < 4.36)					
CONUT score	20/25	1.64 (0.83–3.22)	0.1505		
(>3 vs. ≤ 3)					
2M-RDI	25/20	0.66 (0.34–1.30)	0.2290		
(≥ 66.1% vs. < 66.1%)					
2M-DBR	25/20	0.26 (0.13–0.54)	**0.0003**	0.29(0.11–0.76)	**0.0127**
(≥ 238.9 vs. < 238.9)					

Factors which showed a *p* value less than 0.05 in univariate analysis were used for further multivariate analysis with a step-down procedure. Abbreviations: 2M-DBR, dose intensity/body surface area ratio at 60 days; ALB, albumin; mALBI, modified albumin-bilirubin; BTR, branched-chain amino acid ratio; CONUT, Controlling Nutrition Status; 2M-RDI, relative dose intensity at 60 days; COI, cut-off index; HR, hazard ratio; CI, confidence interval.
